# The effect of recipient BMI on waitlist and post‐transplant outcomes after the 2018 heart transplant allocation policy change

**DOI:** 10.1111/jocs.16432

**Published:** 2022-04-05

**Authors:** Jay N. Patel, David G. Rabkin, Brett W. Sperry, Anju Bhardwaj, Joshua S. Chung, Dmitry Abramov

**Affiliations:** ^1^ Division of Cardiology Loma Linda Veterans Administration Healthcare System Loma Linda California USA; ^2^ Department of Cardiothoracic Surgery Loma Linda University Medical Center Loma Linda California USA; ^3^ Saint Luke's Mid America Heart Institute Kansas City Missouri USA; ^4^ Department of Advanced Cardiopulmonary, Therapies and Transplantation The University of Texas–Houston, Houston Texas USA; ^5^ Department of Medicine, Division of Cardiology Loma Linda University Medical Center Loma Linda California USA

**Keywords:** allocation system, body mass index, heart transplant, mechanical support, post‐transplant outcomes, waitlist outcomes

## Abstract

**Objective:**

The effects of recipient body mass index (BMI) on waitlist strategies, waitlist outcomes, and post‐transplant outcomes among adult patients listed for heart transplantation under the updated 2018 allocation system have not been well characterized.

**Methods:**

The United Network of Organ Sharing data set between October 2015 and March 2021 was analyzed, and patients were grouped based on recipient BMI and whether listing occurred in the old (pre‐October 2018) or new allocation system.

**Results:**

Listing strategies differed by BMI group, but trends of increased use of temporary mechanical support and decreased use of durable support remained among all BMI groups, except those with BMI > 35 kg/m^2^. Waitlist outcomes improved among all BMI cohorts in the new allocation system, including among patients with BMI 30–34.9 and >35 kg/m^2^, although patients with higher BMIs continued to have longer waitlist times. Post‐transplant outcomes in the new allocation system are worse for patients with BMI > 30 kg/m^2^ (hazard ratio: 1.47; confidence interval: 1.19–1.82; *p* < .001).

**Conclusions:**

The 2018 change to the heart transplant allocation system was associated with similar changes in the use of mechanical support for listing strategy across BMI ranges, except in the most obese, and improved waitlist outcomes across all BMI ranges. Post‐transplant outcomes in the new allocation system are worse for patients with BMI > 30 kg/m^2^ compared to patients with BMI < 30 kg/m^2^. These findings have important clinical implications for our understanding of the ongoing influence of BMI on waitlist courses and post‐transplant outcomes among patients listed for heart transplantation

## 1 INTRODUCTION

Body size is an important factor among advanced heart failure patients being evaluated and listed for heart transplantation. Body mass index (BMI) has previously been shown to affect transplant listing strategy,^1^ and was associated with increased waitlist mortality,^1^, ^2^ longer wait times before transplantation,^2^ and decreased post‐transplant survival.^2^, ^3^, ^4^ In 2018, the United Network of Organ Sharing (UNOS) provided significant changes to the heart transplant allocation system, which resulted in changes in listing strategies (increase in temporary mechanical support and decrease in durable ventricular assist devices), decrease in waitlist times, and variable effects on post‐transplant survival.^5^, ^6^, ^7^, ^8^, ^9^, ^10^, ^11^ BMI may have significant effects on decisions regarding listing strategy and the use of mechanical support, which may affect waitlist times and outcomes. As the impact of BMI on these outcomes under the new allocation system have not been well characterized, we sought to evaluate the role of BMI on listing strategy, waitlist outcomes, and post‐transplant outcomes in the current era.

## 2 METHODS

The UNOS data set for all patients >18 years of age between October 2015 and March 2021 was analyzed and grouped based on listing in the old (pre‐October 2018) or new (post‐October 2018) allocation system. Patient demographics, comorbidities, clinical characteristics, and listing status were obtained at the time of listing and donor characteristics were obtained at the time of transplant. Listed patients were followed until one of three competing waitlist outcomes: transplantation, waitlist death, or waitlist removal. Patients who remained on the waitlist at the end of the study period were censored. To avoid bias from changing patterns occurring in anticipation of the allocation change as well as to ensure equal follow‐up, patients listed in the year before the allocation change were excluded. To avoid bias among post‐transplant outcomes, patients with less than 1 year of follow‐up were excluded. Patient characteristics, comorbidities, listing strategies, and outcomes were compared before and after the UNOS allocation change based on BMI at listing. A two‐sample *t* test was used to compare continuous variables and *χ* ^2^ test for categorical variables. Fine–Gray proportional subhazard models were used to estimate the effect of allocation change on competing waitlist outcomes—transplantation, death, or removal from waitlist—among each BMI category, which were the primary outcomes. Unadjusted and adjusted subhazard ratios (SHR) were reported for each competing outcome; multivariable regression models were adjusted for clinical characteristics including age, gender, BMI, education, region, listing strategies, other available comorbidities, and hemodynamics. Cox proportional hazards model was used to evaluate post‐transplant outcomes of survival and retransplantation, which was the secondary outcome.

## 3 RESULTS

Of the total cohort, 7035 patients meeting inclusion criteria were listed for transplant under the old allocation system and 6965 were listed in the new allocation system. Table 1 demonstrates key patient demographics, comorbidities, and listing strategies among each BMI category before and after the new allocation system. Under the old allocation system, 26.6% and 7.7% were listed with BMI of 30–34.9 and >35 kg/m^2^, respectively, while under the new allocation system, 27.0% and 9.1% had those BMI ranges. There were no clinically relevant differences in baseline patient characteristics before and after the UNOS allocation system change. With respect to transplant listing strategies, the new allocation system resulted in more patients being listed with intra‐aortic balloon pump (IABP) and extracorporeal membrane oxygenation, and less patients being listed with inotropic support and durable left ventricular assist device (LVAD). These changes were generally consistent among each BMI category, although patients listed with >35 kg/m^2^ were equally likely to be listed with a durable LVAD under the old and new allocation systems. Under the new allocation system, compared to patients with BMI < 30 kg/m^2^, those with a BMI of >30 kg/m^2^ were less likely to be listed with inotropes (24.6% vs. 34.8%, *p* < .001) or with IABP (10.1% vs. 15.0%, *p* ≤ .001) and more likely to be listed with a durable LVAD (35.9% vs. 24.1%, *p* < .001).

**Table 1:** Patient characteristics among the whole cohort and characterized by BMI in the old and new allocation.

Old UNOS cohort listed: 10/18/15–10/17/17 vs. new UNOS cohort listed: 10/18/18–10/17/20	All		*p *Value	BMI < 25.0		*p *Value	BMI 25.0–29.9		*p* Value	BMI 30.0–34.9		*p* Value	BMI 35.0 and above		*p* Value
Candidate characteristics at listing registration	Old UNOS	New UNOS		Old UNOS	New UNOS		Old UNOS	New UNOS		Old UNOS	New UNOS		Old UNOS	New UNOS	
n (%) or mean (SD)	7035	6965		2137	2022		2489	2436		1869	1876		540	631	
Age at listing	53.2 (12.5)	53.0 (52.7)	.262	52.5 (14.1)	52.3 (14.7)	.551	54.9 (11.8)	54.7 (11.9)	.658	52.9 (11.3)	52.8 (11.6)	.790	49.7 (12.3)	49.3 (11.8)	.570
Male gender	5192 (73.8)	5114 (73.4)	.612	1439 (67.3)	1375 (68.0)	.647	1936 (77.8)	1903 (78.1)	.775	1409 (75.4)	1385 (73.8)	.273	408 (75.6)	451 (71.5)	.115
Diabetes	1977 (28.1)	1958 (28.1)	.973	399 (18.7)	269 (18.3)	.721	687 (27.6)	706 (29.0)	.262	677 (36.2)	634 (33.8)	.119	214 (39.6)	249 (39.5)	.953
Dialysis	108 (1.5)	141 (2.0)	**.028**	39 (1.8)	40 (2.0)	.719	26 (1.0)	50 (2.1)	**.004**	35 (1.9)	39 (2.1)	.650	8 (1.5)	12 (1.9)	.58
Prior stroke	427 (6.1)	487 (7.0)	**.027**	146 (6.8)	159 (7.9)	.204	155 (6.2)	160 (6.6)	.614	97 (5.2)	128 (6.8)	**.035**	29 (5.4)	40 (6.3)	.483
Prior malignancy	619 (8.8)	610 (8.8)	.938	218 (10.2)	196 (9.7)	.581	216 (8.7)	218 (9.0)	.724	151 (8.1)	152 (8.1)	.979	34 (6.3)	44 (7.0)	.643
History of cigarette use	3189 (45.3)	3013 (43.3)	**.015**	865 (40.5)	804 (39.8)	.63	1205 (48.4)	1030 (42.4)	**<.001**	871 (46.6)	885 (47.2)	.726	248 (45.9)	294 (46.6)	.820
Prior cardiac surgery (nontransplant)	2773 (39.4)	2715 (39.0)	.611	791 (37.0)	662 (32.7)	**.004**	1009 (40.6)	984 (40.5)	.956	754 (40.3)	777 (41.4)	.503	219 (40.6)	292 (46.3)	**.049**
Implantable cardiac defbrillator	5376 (76.4)	4960 (71.3)	**<.001**	1534 (71.8)	1318 (65.2)	**<.001**	1914 (76.9)	1749 (72.0)	**<.001**	1486 (79.5)	1421 (75.8)	**.006**	442 (81.9)	472 (74.8)	**.004**
Creatinine	1.27 (0.69)	1.33 (0.95)	**<.001**	1.19 (0.63)	1.22 (0.81)	.307	1.27 (0.61)	1.39 (1.13)	**<.001**	1.35 (0.80)	1.36 (0.82)	.717	1.35 (0.82)	1.37 (0.92)	.651
No support	2473 (35.2)	2546 (36.6)	.084	721 (33.7)	741 (36.7)	.050	909 (36.5)	894 (36.7)	.896	678 (36.3)	696 (37.1)	.601	165 (30.6)	215 (34.1)	.200
Inotropes	2287 (32.5)	2167 (31.1)	.076	877 (41.0)	787 (38.9)	.164	741 (29.8)	764 (31.4)	.225	515 (27.6)	472 (25.2)	.096	154 (28.5)	144 (22.8)	**.026**
IABP	376 (5.3)	921 (13.2)	**<.001**	151 (7.1)	352 (17.4)	**<.001**	117 (4.7)	316 (13.0)	**<.001**	76 (4.1)	197 (10.5)	**<.001**	32 (5.9)	56 (8.9)	.056
Durable LVAD	2265 (32.2)	1974 (28.4)	**<.001**	517 (24.2)	386 (19.1)	**<.001**	828 (33.3)	689 (28.3)	**<.001**	695 (37.2)	643 (34.3)	.063	225 (41.7)	256 (40.6)	.704
Temporary LVAD	100 (1.4)	108 (1.6)	.526	35 (1.6)	38 (1.9)	.555	35 (1.4)	37 (1.5)	.736	20 (1.1)	25 (1.3)	.461	10 (1.9)	8 (1.3)	.418
ECMO	107 (1.5)	212 (3.0)	**<.001**	45 (2.1)	68 (3.4)	**.013**	28 (1.1)	73 (3.0)	**<.001**	23 (1.2)	50 (2.7)	**.001**	11 (2.0)	21 (3.3)	.177
Ventilator support at listing	117 (1.7)	131 (1.9)	.329	42 (2.0)	42 (2.1)	.798	29 (1.2)	41 (1.7)	.125	33 (1.8)	34 (1.8)	.914	13 (2.4)	14 (2.2)	.830
Waitlist outcomes for listed patients—follow‐up censored at 1 year															
Median days on waitlist (Wilcoxon's rank‐sum test)	151 (40–365)	66 (13–340)	**<.001**	96 (26–321)	32 (8–187)	**<.001**	149 (44–365)	65 (12–336)	**<.001**	207 (63–365)	120 (19–365)	**<.001**	238 (63–365)	148 (24–365)	**<.001**
Median days to transplantation	102 (30–286)	26 (8–118)	**<.001**	67 (20–210)	18 (6–70)	**<.001**	110 (34–293)	27 (8–121)	**<.001**	138 (42–328)	38 (11–175)	**<.001**	150 (44–365)	38 (10–160)	**<.001**
Transplanted	3854 (54.8)	4369 (62.7)		1308 (61.2)	1407 (69.6)		1413 (56.8)	1533 (62.9)		907 (41.9)	1094 (58.3)		226 (41.9)	335 (53.1)	
WL death	322 (4.6)	203 (2.9)		95 (4.5)	60 (3.0)		100 (4.0)	61 (2.5)		87 (4.7)	62 (3.3)		40 (7.4)	20 (3.2)	
Removed from waitlist	858 (12.2)	741 (10.6)		252 (11.8)	215 (10.6)		299 (12.0)	267 (11.0)		238 (12.7)	189 (10.1)		69 (12.8)	70 (11.1)	
Remains on waitlist	2001 (28.4)	1652 (23.7)		482 (22.6)	340 (16.8)		677 (27.2)	575 (23.6)		637 (34.1)	531 (28.3)		205 (38.0)	206 (32.7)	
Transplantation rate (per 10,000 patient‐days) (95 CI)	29.9 (29.0–30.9)	43.8 (42.5–45.1)		39.8 (37.7–42.0)	63.2 (60.0–66.6)		31.0 (29.4–32.6)	44.3 (42.1–46.6)		23.5 (22.0–25.0)	34.6 (32.6–36.7)		19.5 (17.1–22.2)	29.7 (26.7–33.1)	
Transplantation rate ratio (95 CI)	1.46 (1.40–1.53)		**<.001**	1.59 (1.47–1.71)		**<.001**	1.43 (1.33–1.54)		**<.001**	1.48 (1.35–1.61)		**<.001**	1.52 (1.28–1.81)		**<.001**
Death rate (per 10,000 patient‐days)	2.5 (2.2–2.8)	2.0 (1.8–2.3)		2.9 (2.4–3.5)	2.7 (2.1–3.5)		2.2 (1.8–2.7)	1.8 (1.4–2.3)		2.2 (1.8–2.8)	2.0 (1.5–2.5)		3.5 (2.5–4.7)	1.8 (1.1–2.7)	
Death rate ratio (95 CI)	0.81 (0.68–0.97)		**.021**	0.93 (0.66–1.30)		.672	0.80 (0.58–1.12)		.180	0.87 (0.62–1.22)		.414	0.51 (0.28–0.90)		**.013**
Competing‐risks regression SHR—transplantation															
Unadjusted	1.41 (1.35–1.47)		**<.001**	1.45 (1.35–1.56)		**<.001**	1.36 (1.27–1.46)		**<.001**	1.46 (1.33–1.59)		**<.001**	1.51 (1.28–1.79)		**<.001**
Adjusted for demographics, hemodynamics, comorbidities, and listing strategy	1.46 (1.40–1.53)		**<.001**	1.54 (1.42–1.66)		**<.001**	1.43 (1.33–1.55)		**<.001**	1.45 (1.32–1.59)		**<.001**	1.58 (1.33–1.89)		**<.001**
Competing‐risks regression SHR—death															
Unadjusted	0.63 (0.53–0.75)		**<.001**	0.66 (0.48–0.92)		**.013**	0.62 (0.45–0.85)		**.003**	0.70 (0.51–0.98)		**.035**	0.42 (0.24–0.71)		**.001**
Adjusted for demographics, hemodynamics, comorbidities, and listing strategy	0.59 (0.49–0.70)		**<.001**	0.61 (0.43–0.86)		**.004**	0.54 (0.38–0.77)		**.001**	0.69 (0.49–0.97)		**.035**	0.36 (0.19–0.65)		**.001**
Competing‐risks regression SHR—WL removal															
Unadjusted	0.87 (0.79–0.96)		**.005**	0.90 (0.75–1.08)		.253	0.92 (0.78–1.08)		.308	0.78 (0.64–0.94)		**.009**	0.87 (0.62–1.21)		.398
Adjusted for demographics, hemodynamics, comorbidities, and listing strategy	0.85 (0.76–0.94)		**.001**	0.87 (0.72–1.05)		.148	0.89 (0.75–1.06)		.190	0.78 (0.64–0.95)		**.013**	0.90 (0.63–1.29)		.562

Old UNOS cohort listed and transplanted: 10/18/15–10/17/17 vs. new UNOS cohort listed and transplanted: 10/18/18–10/17/20	All		*p *Value	BMI < 25.0		*p* Value	BMI 25.0–29.9		*p* Value	BMI 30.0–34.9		*p* Value	BMI 35.0 and above		*p* Value
Recipient characteristics at transplant registration	Old UNOS	New UNOS		Old UNOS	New UNOS		Old UNOS	New UNOS		Old UNOS	New UNOS		Old UNOS	New UNOS	
*n* (%) or mean (SD)	3519	4138		1218	1339		1257	1452		835	1029		209	318	
Age at transplantation	53.9 (12.6)	53.3 (13.0)	**.037**	53.4 (13.8)	52.6 (14.8)	.152	55.4 (12.0)	54.8 (12.1)	.181	52.9 (11.3)	52.8 (11.8)	.863	50.9 (12.3)	50.3 (11.0)	.55
ABO blood group			.124			.229			.523			.203			.058
A	1494 (42.5)	1703 (41.2)		512 (42.0)	544 (40.6)		541 (43.0)	594 (40.9)		353 (42.3)	450 (43.7)		88 (42.1)	115 (36.2)	
B	549 (15.6)	669 (16.2)		202 (16.6)	226 (16.9)		181 (14.4)	224 (15.4)		120 (14.4)	167 (16.2)		46 (22.0)	52 (16.4)	
AB	241 (6.9)	242 (5.9)		81 (6.7)	68 (5.1)		89 (7.1)	93 (6.4)		58 (7.0)	51 (5.0)		13 (6.2)	30 (9.4)	
O	1235 (35.1)	1524 (36.8)		423 (34.7)	501 (37.4)		446 (35.5)	541 (37.3)		304 (36.4)	361 (35.1)		62 (29.7)	121 (38.1)	
Hospitalization status			**<.001**			**<.001**			**<.001**			**<.001**			**<.001**
In ICU	1367 (28.9)	2619 (55.7)		455 (37.4)	875 (65.4)		405 (32.2)	837 (57.6)		243 (29.1)	570 (55.4)		53 (25.4)	156 (49.1)	
Hospitalized—not in ICU	773 (16.3)	565 (12.0)		214 (17.6)	142 (10.6)		185 (14.7)	152 (10.5)		116 (13.9)	116 (11.3)		47 (22.5)	54 (17.0)	
Not Hospitalized	2599 (54.8)	1518 (32.3)		549 (45.1)	322 (24.1)		667 (53.1)	463 (31.9)		476 (57.0)	343 (33.3)		109 (52.2)	108 (34.0)	
No support	430 (12.2)	655 (15.8)	**<.001**	153 (12.6)	198 (14.8)	.102	165 (13.1)	228 (15.7)	.058	96 (11.5)	175 (17.0)	**.001**	16 (7.7)	54 (17.0)	**.002**
Inotropes	1455 (41.4)	1785 (43.1)	.114	589 (48.4)	693 (51.8)	.086	504 (40.1)	612 (42.2)	0.279	297 (35.6)	374 (36.4)	.728	65 (31.1)	106 (33.3)	.592
IABP	337 (9.6)	1364 (33.0)	**<.001**	133 (10.9)	513 (38.3)	**<.001**	111 (8.8)	467 (32.2)	**<.001**	78 (9.3)	306 (29.7)	**<.001**	15 (7.2)	74 (24.5)	**<.001**
Durable LVAD	1473 (41.9)	1180 (28.5)	**<.001**	400 (32.8)	278 (20.8)	**<.001**	546 (43.4)	416 (28.7)	**<.001**	404 (48.4)	355 (34.5)	**<.001**	123 (58.9)	131 (41.2)	**<.001**
Temporary LVAD	106 (3.01)	115 (2.8)	.544	32 (2.6)	35 (2.6)	0.983	38 (3.0)	38 (2.6)	.523	27 (3.2)	32 (3.1)	.879	9 (4.3)	10 (3.1)	.484
ECMO	37 (1.05)	247 (6.0)	**<.001**	15 (1.2)	82 (6.1)	**<.001**	11 (0.9)	88 (6.1)	**<.001**	9 (1.1)	54 (5.3)	**<.001**	2 (1.0)	23 (7.2)	**.001**
Ventilator support	38 (1.1)	100 (2.4)	**<.001**	17 (1.4)	34 (2.5)	**.039**	10 (0.8)	36 (2.5)	**<.001**	8 (1.0)	21 (2.0)	.060	3 (1.4)	9 (2.8)	.294
Creatinine	1.25 (0.92)	1.21 (0.60)	**.024**	1.21 (1.28)	1.13 (0.77)	.065	1.25 (0.77)	1.22 (0.44)	.138	1.29 (0.49)	1.28 (0.56)	.588	1.29 (0.43)	1.27 (0.53)	.700
Total bilirubin	0.93 (1.47)	1.05 (2.09)	**.004**	1.04 (1.94)	1.05 (1.71)	.897	0.85 (0.86)	1.03 (2.25)	**.007**	0.87 (1.41)	1.10 (2.49)	**.0192**	0.97 (1.39)	0.98 (1.05)	.941
Cardiac hemodynamics															
PA systolic	39.3 (13.5)	40.5 (13.3)	**<.001**	39.2 (13.6)	40.4 (13.1)	**.025**	39.0 (13.6)	40.4 (13.4)	**.007**	39.8 (13.1)	40.9 (13.5)	.092	39.4 (13.2)	39.7 (13.7)	.804
PA systolic	18.8 (8.2)	19.9 (8.5)	**<.001**	18.7 (8.2)	19.5 (8.0)	**.006**	18.5 (8.1)	19.7 (8.4)	**<.001**	19.2 (8.2)	20.6 (8.9)	**.001**	19.1 (8.9)	19.7 (9.1)	.485
Mean PA	26.7 (9.6)	27.7 (9.8)	**<.001**	26.5 (9.8)	27.5 (9.5)	**.009**	26.4 (9.5)	27.5 (9.7)	**.002**	27.3 (9.4)	28.3 (10.2)	**.037**	26.6 (10.0)	27.4 (10.4)	.411
Mean PCWP	17.4 (8.5)	18.4 (8.4)	**<.001**	17.6 (8.8)	18.3 (8.2)	**.032**	17.0 (8.3)	18.2 (8.3)	**<.001**	17.7 (8.2)	18.8 (8.7)	**.007**	17.7 (8.7)	18.2 (8.7)	.443
CO	4.51 (1.41)	4.35 (1.45)	**<.001**	4.04 (1.23)	3.98 (1.34)	.294	4.56 (1.37)	4.38 (1.37)	**<.001**	4.91 (1.44)	4.62 (1.40)	**<.001**	5.31 (1.42)	4.98 (1.54)	**.014**

*Note*: Bold values are statistical significance at *p* < 0.05.

Abbreviations: BMI, body mass index; CI, confidence interval; CO, cardiac output; ECMO, extracorporeal membrane oxygenation; IABP, intra‐aortic balloon pump; ICU, intensive care unit; IQR, interquartile range; LV, left ventricular; LVAD, left ventricular assist device; PA, pulmonary artery; PCWP, pulmonary capillary wedge pressure; SHR, subhazard ratio; UNOS, United Network of Organ Sharing; WL, waitlist.

**Figure 1:**
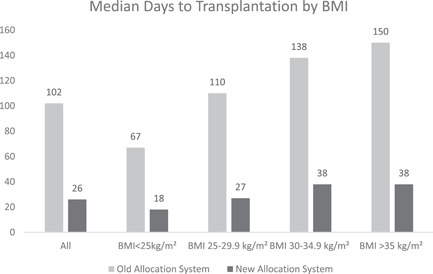
Median days to transplantation among the whole cohort and characterized by body mass index in the old and new allocation systems.

Table 1 also demonstrates the effects of the allocation change on waitlist outcomes by BMI group. The new allocation system was associated with a reduction in waitlist mortality and waitlist removal, and improved transplantation rates among all BMI groups. However, under the new allocation system, patients with BMI > 30 kg/m^2^ continued to have higher median days to transplantation (38 vs. 21 days, *p* < .001), and lower unadjusted (hazard ratio [HR]: 0.75; confidence interval [CI]: 0.70–0.80; *p* < .001) and adjusted (HR: 0.83; CI: 0.77–0.89; *p* < .001) SHRs for transplantation compared to patients with BMI < 30 kg/m^2^. Among patients in the new allocation system with BMI > 30 kg/m^2^, those with BMI > 35 kg/m^2^ had similar median days to transplantation, 38 (11–175) vs. 38 (10–160), *p* = .754, and similar days on the waitlist, 148 (24–365) vs. 120 (19–365), *p* = .108 compared to patients with BMI between 30 and 35 kg/m^2^. Figure 1 highlights the differences in the median days to transplantation based on BMI between the old and new allocation systems.

Table 2 demonstrates donor characteristics among the whole cohort as well as based on recipient BMI. Male donors were more common among recipients with higher BMI. There was an increase in ischemic time and travel distance in the new allocation system, although the increases were similar among BMI cohorts. There were no differences in donor BMI among any cohort between the allocation systems.

**Table 2:** Donor and transplant characteristics among the whole cohort and characterized by BMI in the old and new allocation systems.

Old UNOS cohort listed and transplanted: 10/18/15–10/17/17 vs. new UNOS cohort listed and transplanted: 10/18/18–10/17/20	All		*p* Value	BMI < 25.0		*p* Value	BMI 25.0–29.9		*p* Value	BMI 30.0–34.9		*p* Value	BMI 35.0 and above		*p* Value
Donor characteristics at transplant registration	Old UNOS	New UNOS		Old UNOS	New UNOS		Old UNOS	New UNOS		Old UNOS	New UNOS		Old UNOS	New UNOS	
*n* (%) or mean (SD)	3519	4138		1218	1339		1257	1452		835	1029		209	318	
Donor age	32.1 (11.1)	32.2 (10.5)	.544	31.7 (11.5)	31.4 (10.7)	.400	32.2 (11.1)	32.4 (10.7)	.559	32.3 (10.5)	32.4 (10.0)	.817	32.5 (10.7)	34.3 (10.6)	.058
Donor male gender	2341 (66.5)	2980 (72.0)	**<.001**	695 (57.1)	903 (67.4)	**<.001**	873 (69.5)	1056 (72.7)	.060	607 (72.7)	779 (75.7)	.139	166 (79.4)	242 (76.1)	.372
Recipient–donor gender mismatch	856 (24.3)	919 (22.2)	**.029**	362 (29.7)	314 (23.5)	**<.001**	284 (22.6)	330 (22.7)	.934	172 (20.6)	202 (19.6)	.604	38 (18.2)	73 (23.0)	.189
Donor body mass index	27.6 (6.4)	27.9 (6.2)	.101	26.0 (5.9)	26.1 (5.4)	.424	27.5 (6.2)	27.7 (6.0)	.577	29.5 (6.4)	29.5 (6.7)	.845	30.6 (7.3)	30.7 (6.9)	.882
Diabetes	165 (3.5)	172 (3.6)	.692	38 (3.1)	42 (3.1)	.984	41 (3.3)	47 (3.2)	.968	34 (4.1)	42 (4.1)	.992	9 (4.3)	15 (4.7)	.825
History of cocaine use	1156 (24.4)	1282 (27.2)	**.002**	270 (22.2)	358 (26.8)	**.004**	288 (23.0)	397 (27.4)	**.026**	222 (26.6)	289 (28.1)	.706	61 (29.2)	92 (28.9)	.385
Creatinine	1.52 (1.58)	1.67 (1.78)	**<.001**	1.37 (1.36)	1.56 (1.67)	**.001**	1.54 (1.69)	1.70 (1.81)	**.018**	1.66 (1.65)	1.68 (1.72)	.790	1.72 (1.74)	2.00 (2.22)	.126
Total bilirubin	1.03 (1.20)	1.04 (1.46)	.599	1.04 (1.34)	1.03 (1.40)	.929	1.00 (1.13)	1.10 (1.63)	.064	1.05 (1.14)	1.01 (1.35)	.469	1.06 (1.02)	0.96 (1.22)	.313
LV ejection fraction	61.6 (6.6)	61.5 (6.8)	.407	61.6 (6.7)	61.4 (6.7)	.376	61.7 (6.5)	61.7 (6.9)	.914	61.4 (6.3)	61.5 (6.6)	.732	62.1 (7.1)	61.2 (7.2)	.146
Ischemic time	3.06 (1.05)	3.45 (1.07)	**<.001**	3.08 (1.03)	3.42 (1.03)	**<.001**	3.07 (1.09)	3.41 (1.07)	**<.001**	2.99 (1.04)	3.49 (1.04)	**<.001**	3.09 (0.97)	3.62 (1.29)	**<.001**
Distance (miles) from Tx center, median (IQR)	72 (12–144)	231 (86–405)	**<.001**	84 (13–291)	230 (84–405)	**<.001**	80 (12–250)	224 (79–394)	**<.001**	50 (8–192)	234 (101–405)	**<.001**	70 (15–238)	251 (104–439)	**<.001**

*Note*: Bold values are statistical significance at *p* < 0.05.

Abbreviations: BMI, body mass index; IQR, interquartile range; UNOS, United Network of Organ Sharing.

Regarding post‐transplant outcomes, when accounting for equal follow‐up, there were no differences between outcomes in the old versus new allocation system among the whole cohort as well as among the studied BMI cohorts (Table S1^1^). However, post‐transplant outcome of death or retransplantation was similar among patients with BMI > 30 kg/m^2^ compared to BMI < 30 kg/m^2^ in the old UNOS (HR: 1.22; CI: 0.96–1.56; *p* = .101), but significantly higher in the new UNOS system (HR: 1.47; CI: 1.19–1.82; *p* < .001). Among patients in the new system with BMI > 30 kg/m^2^, those with BMI > 35 kg/m^2^ had similar rates of post‐transplant adverse events compared to the BMI group of 30–35 kg/m^2^ (HR: 1.15; CI: 0.79–1.57; *p* = .457).

## 4 DISCUSSION

The present work demonstrates several important findings. Changes in listing strategies associated with the new allocation system were similar across BMI ranges, although differences between the systems were less prominent among patients with BMI of >35 kg/m^2^. Under the new allocation system, compared to patients with BMI < 30 kg/m^2^, those with a BMI of >30 kg/m^2^ were less likely to be listed with inotropes or with IABP and more likely to be listed with a durable LVAD. Median days on the waitlist decreased significantly in the new allocation system for all BMI ranges, although they remain highest for patients with BMI > 30 kg/m^2^. Finally, post‐transplant outcomes in the new allocation system were similar compared to the old allocation system for all BMI ranges, although when categorized with a BMI cutoff of <30 vs. >30 kg/m^2^, those with BMI of >30 kg/m^2^ experienced the worst outcomes under the new allocation system, but not under the old allocation system. These findings have important clinical implications for our understanding of the ongoing influence of BMI on waitlist course and post‐transplant outcomes among patients listed for heart transplantation.

These findings support prior studies showing an increase in waitlist times^2^ and increase in post‐transplant mortality^3^, ^4^ among patients with obesity, although prior studies demonstrated that the increase in post‐transplant mortality among obese patients is modest and not consistent among all studies. Longer waitlist times for patients with BMI > 30 kg/m^2^ may occur due to the requirement for adequate donor–recipient size matching as well as due to variation in listing strategy where obese patients may be listed at lower status (due to higher use of durable LVAD, higher rate of listing without mechanical/inotropic support, and lower use of IABP). However, the median days to transplantation and transplantation rate for patients with BMI > 30 kg/m^2^ and particularly BMI > 35 kg/m^2^ have improved significantly under the new allocation system and are now better in this population than these parameters were for any BMI under the old allocation system.

The current analysis, when ensuring for equal follow‐up, demonstrates similar 1‐year outcomes after implementation of the new allocation system change among all patients, and extends these findings based on BMI cohorts. However, under the new allocation system, patients with BMI > 30 kg/m^2^ (and particularly those with BMI > 35 kg/m^2^) had higher rates of post‐transplant death or retransplantation. The differences in increased adverse events in patients with BMI > 30 kg/m^2^ are more pronounced after changes in the allocation system, and the current study did not demonstrate this adverse effect of BMI > 30 kg/m^2^ under the old allocation system. Censoring at 1 year to allow for equal follow‐up as well as the inclusion of multivariable analyses in establishing the effects of BMI on outcomes may explain the difference between these findings and prior publications examining the effect of obesity on post‐transplant outcomes.

Taken together, the improved waitlist outcomes with a more pronounced increase in post‐transplant adverse events under the new allocation system have important implications for the care of obese patients being considered for heart transplantation. Improved waitlist outcomes and decreased waitlist times can provide reassurance that transplant remains a feasible strategy among adequately selected patients with obesity, including those with BMI > 35 kg/m^2^. Patients with BMI > 30 kg/m^2^ experience higher post‐transplant adverse events compared to patients with BMI < 30 kg/m^2^. Optimizing BMI and associated risk factors before transplant as well as increased focus on post‐transplant care may therefore be important targets to improve the outcomes in advanced heart failure patients with elevated BMI being considered for transplantation. Additional analyses will be needed to explore the contributors to increased adverse post‐transplant outcomes among those with higher BMI, particularly as noted in the new allocation system.

There are limitations to this study, including those which are inherent to the use of large data sets for analyses. The UNOS data set is reliant on entry from individual transplant centers and lacks important information that may affect decision making surrounding the care of patients based on their BMI. Patients who were not listed for transplant because of their BMI or offered destination therapy LVAD options as opposed to transplant listing are not evaluated in this study.

## 5 CONCLUSION

In summary, the 2018 change to the UNOS allocation system resulted in changes in waitlist management and improved waitlist outcomes that were generally similar based on BMI cohorts, including among patients with BMI of 30–35 kg/m^2^. Patients with BMI > 30 kg/m^2^ experience worse post‐transplant outcomes compared to patients with BMI < 30 kg/m^2^ under the new allocation system.

## CONFLICTS OF INTEREST

The authors declare no conflicts of interest.

## Supporting information

Supplementary information.
